# Pregnancy and risk of renal cell cancer: a population-based study in Sweden

**DOI:** 10.1038/sj.bjc.6600263

**Published:** 2002-05-06

**Authors:** M Lambe, P Lindblad, J Wuu, R Remler, C-c Hsieh

**Affiliations:** Department of Medical Epidemiology, Karolinska Institutet, Stockholm, Sweden; Department of Urology, Sundsvall Hospital, Sundsvall, Sweden; Cancer Center, University of Massachusetts Medical School, Worcester, Massachusetts, USA; Division of Public Health Sciences, Fred Hutchinson Cancer Research Center, Seattle, Washington, USA; Department of Epidemiology, Harvard School of Public Health, Boston, Massachusetts, USA

**Keywords:** kidney neoplasms, risk factors, parity, pregnancy, case–control studies

## Abstract

Epidemiological findings indicate that hormonal influences may play a role in the etiology of renal cell cancer (RCC). The possible effect of childbearing remains enigmatic; while some investigators have reported a positive association between number of births and renal cell cancer risk, others have not. A case–control study, nested within a nation-wide Fertility Register covering Swedish women born 1925 and later, was undertaken to explore possible associations between parity and age at first birth and the risk of renal cell cancer. Among these women a total of 1465 cases of RCC were identified in the Swedish Cancer Register between 1958 and 1992 and information on the number of live childbirths and age at each birth was obtained by linkage to the Fertility Database. For each case, five age-matched controls were randomly selected from the same register. Compared to nulliparous women, ever-parous women were at a 40% increased risk of RCC (Odds Ratio [OR]=1.42; 95% CI 1.19-1.69). The corresponding OR for women of high parity (five or more live births) was 1.91 (95% CI 1.40–2.62). After controlling for age at first birth among parous women, each additional birth was associated with a 15% increase in risk (OR=1.15; 95% CI 1.08–1.22). The observed positive association between parity and renal cell cancer risk is unlikely to be fully explained by uncontrolled confounding, but warrants further evaluation in large studies, with allowance for body mass index.

*British Journal of Cancer* (2002) **86**, 1425–1429. DOI: 10.1038/sj/bjc/6600263 www.bjcancer.com

© 2002 Cancer Research UK

## 

Cancer of the renal parenchyma, or renal cell cancer (RCC), represents about 80% of all kidney cancer cases in Sweden and is about twice as common in men than among women. Incidence rates in Sweden have tended to decrease among both men and women (Swedish Cancer Registry, 2000), during the past two decades in contrast to many other areas in the world. Reported international incidence rates vary more than 10-fold, with the highest rates found in northern, western and eastern Europe, Australia and North America (Ferlay *et al*, 2001). Apart from cigarette smoking and obesity, and possibly weight cycling, hypertension and/or antihypertensive medication, the causes of renal cell cancer are unclear (McLaughlin and Lipworth, 2000).

Experimental findings of oestrogen-induced renal cell carcinogenesis in animals (Li and Li, 1990) have prompted an interest in the possible influence of hormonal factors in humans. Weak positive associations have been reported for use of oral contraceptives (McLaughlin *et al*, 1992) and replacement oestrogens in some (Asal *et al*, 1988; McLaughlin *et al*, 1992), but not all studies (McLaughlin *et al*, 1984; Adami *et al*, 1998). A large case–control study, however, found a reduced risk of renal cell cancer following oral contraceptive use (Lindblad *et al*, 1995), although another recent study found no relationship (Gago-Dominguez *et al*, 1999). Two studies showed no relation between hysterectomy or oophorectomy and risk of RCC (Wynder *et al*, 1974; McLaughlin *et al*, 1984), whereas other investigators have reported an increased risk following hysterectomy (McCredie and Stewart, 1992; Mellemgaard *et al*, 1994; Gago-Dominguez *et al*, 1999) and both hysterectomy and oophorectomy (Lindblad *et al*, 1995). There is also some epidemiological evidence that childbearing may play a role in the development of RCC (Kreiger *et al*, 1993; Chow *et al*, 1995; Lindblad *et al*, 1995).

To further examine this issue, we utilised a large population-based data set to explore possible associations between pregnancy and the risk of renal cell cancer among Swedish women.

## MATERIALS AND METHODS

We merged data from two population-based Swedish registers, the Fertility Register and the Cancer Register, both updated through 1992, through a unique national registration number.

The Fertility Register was originally based on women born between 1925 and 1960 who were resident citizens of Sweden in 1960. For these women, reproductive data (nulliparity, number and dates of live births) during the period 1943–1960 were collected retrospectively at the 1960 Census. Later birth cohorts of all Swedish women have been added continuously, with the women's births being recorded annually via vital statistics records. For the present study, the Fertility Register included almost 3.4 million live births recorded from 1943 to 1992 among approximately 2.4 million women born between 1925 and 1972. Based on a nation-wide reporting system of all births to women residing in Sweden, the quality of data concerning number and dates of birth is generally high (Johansson and Finnäs, 1983). In the oldest cohorts, mainly women born 1925–29, fertility may be underestimated since children born before 1943, those not living with their mothers, and those who died before the 1960 Census, were not recorded. There might also be a slight overestimation of fertility between 1943 and 1960 because adopted and foster children were included in the retrospectively collected information. From 1961 and onwards, only biological children were included. Dates of death and emigration among registered women are recorded yearly in the Fertility Register. Because women with 10 or more births are rare in the birth cohorts under study, complete birth dates were only obtained for up to nine children per woman for the purpose of this study. Subjects with 10 or more births were identifiable by a two-digit parity code.

The Swedish Cancer Register started in 1958. All newly diagnosed malignant tumours must be reported to the Register separately by both the clinician and the pathologist or cytologist. Nearly 100% of all diagnosed cancers are recorded (Swedish Cancer Registry, 2000). Among resident Swedish women born between 1925 and 1972, a total of 2086 cases of kidney cancer (International Classification of Diseases (ICD)-7 : 180) were recorded in the Cancer Register from January 1, 1958 to December 31, 1992. Of these, 1856 (88.9 %) were identified in the Fertility Register. Thus, no match was achieved for 230 cases recorded in the Cancer Register, some of which represent mismatches due to aberrant national registration numbers, while the majority are non-Swedish citizens who are recorded only in the Cancer Register. We excluded 14 cases diagnosed in 1958–1960 who were survivors with prevalent cancers at the time of the 1960 Census, two cases with more than nine children, 193 cases with non-renal cell cancer and 368 cases with histopathological-codes other than adenocarcinoma (code: 096). Because of overlap between the excluded groups, the final analysis encompassed 1465 cases of renal cell cancer with concomitant fertility information.

A nested case–control study was undertaken to evaluate possible associations between the recorded reproductive variables and RCC. For every case, five comparison women (controls) matched on year and month of birth were randomly selected among those in the Fertility Register. The controls had to be alive at the date of the diagnosis of the case and be residents of Sweden, without themselves having been diagnosed with kidney cancer. Controls were assigned an index date that was the same as the diagnosis date of their matched case. For both cases and controls, only pregnancies before the date of the diagnosis of the case (the index date for the control) were included in the analysis. Controls with more than nine births were excluded, leaving five case–control sets being matched 1:4.

### Statistical analysis

The statistical analyses used matched conditional logistic regression (Breslow and Day, 1980). Associations between the reproductive risk factors and RCC were measured using odds ratios (OR) as estimates of relative risks (Rothman and Greenland, 1998), and their 95% confidence intervals (CI). Age at first birth was analysed both as a categorical variable (nulliparous, <20, 20–24, 25–29, 30+) and as a continuous variable in the analysis for parous women. Risk estimates with time since most recent pregnancy were calculated among uniparous, biparous and triparous women, compared to nulliparous women.

## RESULTS

More than half of all renal cell cancers (59.9%) in women were diagnosed at age 50 or over (Table 1

**Table 1:**
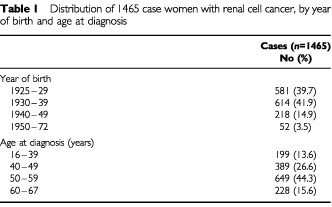
Distribution of 1465 case women with renal cell cancer, by year of birth and age at diagnosis

), with a mean age at diagnosis of 50.4 years. We found a strong positive association between number of births and risk of RCC (Table 2

**Table 2:**
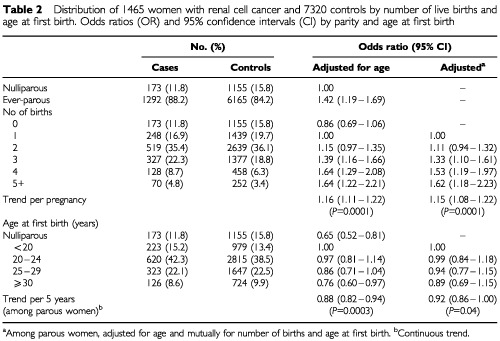
Distribution of 1465 women with renal cell cancer and 7320 controls by number of live births and age at first birth. Odds ratios (OR) and 95% confidence intervals (CI) by parity and age at first birth

). Compared to nulliparous women, ever parous women were at a 40% increased risk of RCC (OR=1.42; 95% CI=1.19–1.69). The corresponding OR for women of high parity (five or more live births) was 1.91 (95% CI=1.40–2.62). After controlling for age at first birth among parous women, risk increased monotonically and each additional birth was associated with a 15% increase in risk (OR=1.15; 95% CI=1.08–1.22). Relative to uniparous women, the adjusted odds ratio for women with three births was 1.33 (95% CI=1.10–1.61) and for women with five or more births the OR was 1.62 (95% CI=1.18–2.23) (Table 2). The positive association with parity was similar in women older at diagnosis (age ⩾50 years) and in those younger at diagnosis (<50 years) (data not shown). When age at first birth was analysed as a continuous variable, there was a slight decrease in risk with increasing age at first delivery. After controlling for parity among parous women, the risk decreased by 8% for each 5-year increment of delay of first birth (OR=0.92; 95% CI=0.86–1.00) (Table 2).

Table 3

**Table 3:**
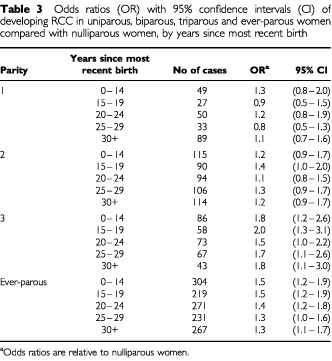
Odds ratios (OR) with 95% confidence intervals (CI) of developing RCC in uniparous, biparous, triparous and ever-parous women compared with nulliparous women, by years since most recent birth

shows the risk of developing RCC by years since most recent birth among uniparous, biparous, triparous and ever-parous women relative to nulliparous women. Except for women with one live birth, risk estimates were consistently and often significantly elevated in all time periods since delivery.

## DISCUSSION

The influence of number of births on the risk of RCC remains unclear with a majority of studies finding no clear relation (Wynder *et al*, 1974; McLaughlin *et al*, 1984, 1992; Cantor *et al*, 1993; La Vecchia *et al*, 1993; Mellemgaard *et al*, 1994; Gago-Dominguez *et al*, 1999), while some investigators have reported positive associations (Kreiger *et al*, 1993; Chow *et al*, 1995; Lindblad *et al*, 1995).

Our results indicate that women with a history of high parity are at an increased risk of renal cell cancer. Compared to nulliparous women, the risk was nearly two times higher among women with five or more live births. This finding is in agreement with that of a large international multi-centre case–control study that found an 80% increased risk among high parity women. However, in that study there was no regular trend with increasing parity. In the same study, age at first birth was not associated with RCC risk after adjustment for age, study centre, body mass index and number of births (Lindblad *et al*, 1995). Compared to women with one or two births, multiparous women showed a more than two-fold risk increase, an association that was stronger among hypertensive or overweight women (Chow *et al*, 1995). In another study, a risk increase of similar magnitude was reported in multiparous women compared to nulliparous women, which was strictly limited to women with a previous history of foetal loss; among women with no foetal loss, the number of live births was unrelated to the risk of RCC (Kreiger *et al*, 1993). The latter study, as in our own, showed evidence of an inverse association between age at first birth and the risk of RCC.

The main strengths of the present study were its large size and the population-based design that minimised selection and information biases. By using nation-wide registry information, we were able to ascertain virtually all incident cases of RCC. The primary weakness was the absence of information on potential confounders such as Body Mass Index (BMI), weight cycling, hypertension, and smoking habits. Furthermore, in our assessment, pregnancy histories on complications or outcomes such as spontaneous and induced abortions were not available.

Of particular concern was the influence on our risk estimates by uncontrolled confounding by BMI. Childbearing is associated with a considerable increase in weight, usually followed by some degree of permanent weight-retention after delivery and lactation (Rössner, 1997). Results from a meta-analysis of 14 studies on obesity and the risk of RCC in women, indicate that the risk increases by 7% for each unit increase in BMI (Bergstrom *et al*, 2001).

Thus, pregnancy-associated weight gain may have influenced the observed relation to parity. To indirectly assess the relative importance of parity and BMI, we obtained data from the Swedish Medical Birth Register, a population-based database that since 1992 records pre-pregnancy weight among expecting mothers. These data showed that pre-pregnancy BMI increased by an average of 3.3 units between a first and sixth pregnancy (Table 4

**Table 4:**
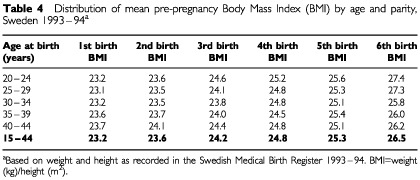
Distribution of mean pre-pregnancy Body Mass Index (BMI) by age and parity, Sweden 1993–94^a^

), producing an expected risk increase for RCC equalling 25.0% (1.07^3.3^). In the present study, we found an estimated risk increase of 62% for women of parity 5+, relative to uniparous women. Taken together, these data provide evidence of an independent risk-modifying effect of parity on the risk of developing RCC, over and beyond the influence of BMI. This assumption is supported by the findings of the collaborative international study on renal cell cancer, where neither adjustment for smoking nor body mass index altered the estimates for parity (Lindblad *et al*, 1995). Chow *et al* (1995) reported an interaction between parity, obesity and the risk of RCC. In that study, the positive association with number of births was present in women with normal weight, but was stronger among obese subjects.

We can only speculate about the biological mechanisms whereby childbearing could affect renal carcinogenesis. Pregnancy-associated hormonal changes, particularly high oestrogen levels, may act as promotors of malignant change by stimulating renal cell proliferation either directly or via paracrine growth factors (Concolino *et al*, 1993). Both oestrogen and progesterone receptors are present in normal and malignant renal cells (Ronchi *et al*, 1984). High doses of potent oestrogens have been shown to induce renal cell tumours in laboratory rodents (Li and Li, 1990). The proliferative effect of oestrogen on kidney tissue seen experimentally may be mediated through the up-regulation of IGF-I receptors (Chen *et al*, 1996). Also, both insulin and IGF-I might contribute to the growth and proliferation of renal cell cancer. Experimentally evidence indicates that such growth factors and their receptors play a role in several renal diseases, including renal tumours (Zumkeller and Schofield, 1992; Stadler and Vogelzang, 1993; Kellerer *et al*, 1995). The consistent finding that obesity is a risk factor for human renal cell cancer, and the knowledge that obesity may lead to a hyperestrogenic environment and increased levels of insulin and insulin-like growth factor (IGF-1) (Azziz, 1989; Frystyk *et al*, 1995), provides indirect support that hormonal factors may play a role in human renal carcinogenesis. Also, epidemiological studies indicate that patients with diabetes have an increased risk of renal cell cancer (Schlehofer *et al*, 1996; Lindblad *et al*, 1999). In addition, a normal pregnancy is associated with hyperfiltration (Krutzen *et al*, 1992; Sturgiss *et al*, 1996). Animal studies suggest that glomerular hyperfiltration could play a role in the development of glomerulosclerosis, (Neuringer and Brenner, 1992; Westenend *et al*, 1992), which makes the nephrons more vulnerable for exposure to carcinogens.

The results of the present study are interesting because of both the highly regular trend between increasing parity and the risk of RCC, and the strength of the associations. Our observations appear unlikely to be fully explained by uncontrolled confounding and warrant further evaluation in large studies where detailed information on other risk factors for RCC and possible confounders also can be considered.
